# The Display Makes a Difference: A Mobile Eye Tracking Study on the Perception of Art Before and After a Museum’s Rearrangement

**DOI:** 10.16910/jemr.13.2.6

**Published:** 2020-06-19

**Authors:** Luise Reitstätter, Hanna Brinkmann, Thiago Santini, Eva Specker, Zoya Dare, Flora Bakondi, Anna Miscená, Enkelejda Kasneci, Helmut Leder, Raphael Rosenberg

**Affiliations:** Department of Art History, University of Vienna, Austria; Department of Computer Science, University of Tübingen, Germany; Faculty of Psychology, University of Vienna, Austria; Vienna Cognitive Science Hub, University of Vienna, Austria; MECS, Leuphana University Lüneburg, Germany

**Keywords:** mobile eye tracking, usability, museum studies, art perception, visitor research, exhibition display, attention, social influences

## Abstract

There is increasing awareness that the perception of art is affected by the way it is presented. In 2018, the Austrian Gallery Belvedere redisplayed its permanent collection. Our multidisciplinary team seized this opportunity to investigate the viewing behavior of specific artworks both before and after the museum’s rearrangement. In contrast to previous mobile eye tracking (MET) studies in museums, this study benefits from the comparison of two realistic display conditions (without any research interference), an unconstrained study design (working with regular museum visitors), and a large data sample (comprising 259 participants). We employed a mixed-method approach that combined mobile eye tracking, subjective mapping (a drawing task in conjunction with an open interview), and a questionnaire in order to relate gaze patterns to processes of meaning-making. Our results show that the new display made a difference in that it 1) generally increased the viewing times of the artworks; 2) clearly extended the reading times of labels; and 3) deepened visitors’ engagement with the artworks in their exhibition reflections. In contrast, interest in specific artworks and art form preferences proved to be robust and independent of presentation modes.

## Introduction

Since the 18^th^ century, with the birth of the public museum, the way art is presented has been crucial to discussions about its perception (1, 2, 3, 4, 5). Today, the umbrella term “display” encompasses the visual, material, and social aspects of art presentations (6, 7, 8, 9). The idea that not only the “what” but also the “how” of an exhibition provides meaning has become a commonplace of contemporary museum and curatorial studies. This new emphasis is also pivotal to research on art perception, as Pelowski, Forster et al. (10) state: “Factors related to the presentational context may mark the most overlooked and potentially most fruitful area for future research on the psychology of art.” But how substantial is the influence of display on our museum experience? And to what extent does display really matter with respect to different viewing patterns? To answer these questions, studies on art perception need to transfer their site of research from the laboratory to the museum itself.

In the art museum, the visual sense is leading: We walk from painting to painting and around sculptures, read textual information, and negotiate our paths in order to set our gaze in place. The gaze, which can be registered with eye tracking, is the bridge between the artworks and us. Soon after the first eye tracking devices were built, artworks were used as stimuli—but by psychologists, not by art historians (11, 12). More recently, the experimental investigation of eye movements was introduced to art history (13). Several studies have used eye trackers to analyze the perception of single artworks ( 14, 15); to test general assumptions from art history (16, 17, 18, 19); or to detect variety and diversity among groups of viewers (13, 20, 21). However, to date, most eye tracking studies on art perception have been conducted in laboratories and with two-dimensional reproductions of artworks. While these studies have delivered remarkable results, they also have severe limitations: The difference between an original artwork and its reproduction is not only referential but essential; moreover, the effect of a museum’s presentational context clearly cannot be studied in a laboratory setting. When moving from the lab to the museum, looking at art is embedded into socio-spatial constellations known to be far more engaging and satisfying ( 22, 23, 24, 25).

Empirical evidence in visitor studies proves that seeing in the museum is everything but static. The combined activity of seeing and moving was already the subject of early visitor studies (26, 27, 28) and continues to be investigated in timing and tracking studies up to the present day, with or without location-sensing technology (29). These studies have provided valuable insights into common viewing times and patterns when looking at art and reading labels (30, 31, 32); for establishing a Sweep Rate Index comparing dwell time in different sorts of exhibitions (33, 34) and for theoretically framing an exhibition visit in an attention-value model (35). Other, more exploratory studies have analyzed visitors’ pathways and experiences in relation to the curatorial design of the exhibition space (36, 37, 38). Methodologically, video recordings shifted the research focus from attention-giving to multi-modal interaction analysis between artworks and co-present subjects (39, 40, 41). Engagement with art is also mirrored in the more general literature on “entrance narratives” as the internal storyline that visitors bring with them (42, 43, 44) to the on-site museum experience (45, 46, 47) in the context of informal and social learning theories (48). Here, visitors are understood as active interpreters who engage with the given content in a structured perception scenario.

The very specific processes that occur in the visitor, i.e. the nature of art perception, are studied in the field of empirical aesthetics. Since Fechner’s preschool of aesthetics (49) and in his tradition, empirical aesthetics have sought to provide more and more detailed descriptions of aesthetic experiences. These models comprise affective and cognitive sub-processes, range from perceptual to higher orders, and explain related cognitive processes (50) as well as affective preferences, emotions, and even transformative processes ( 51). Regarding the value of art, an analysis of the commonalities in aesthetic experiences in museums, and beyond, by Pelowski, Forster et al. (10) has revealed that memory seems common to all psychological approaches. Such studies agree that affective as well as meaning- and appraisal-related processes are essential. Pelowski et al. (52) have also provided a taxonomy of responses to art that they empirically studied for two installations of the artist Ólafur Elíasson. In a pre-post design, with questionnaires and partly also eye tracking, they were able to confirm the expected differences in appraisal strategies, enjoyment, and meaning making.

Current technical developments of psycho-physiological devices are expected to boost visitor research and perception studies. Among these, “eye tracking may prove to be the most powerful tool for museum studies” (53), as it provides precise data on the sensory viewing process. So far, however, eye tracking studies in museums have not reached their full potential. For example, Heidenreich and Turano (54) recorded only four museum visitors at the Baltimore Museum of Art. Quiroga, Dudley, and Binnie (55) studied six participants who viewed a single painting at the Tate Britain. At the Indianapolis Museum of Art, Bachta et al. (56) instructed visitors to sit in front of a remote eye tracker. Brieber, Nadal, Leder, and Rosenberg (22) gave their participants a specific route to follow. Thalwitzer, Brinkmann, and Rosenberg used a calibration-free gaze tracker at the Kunsthistorisches Museum in Vienna in 2015, but with poor data quality ( 57). Wagner (58) explored mobile eye tracking (MET) in regard to sculptures, albeit with only one, non-moving subject. Wiseman et al. ( 59) combined MET with motion capture to investigate how vision and body interact when perceiving a sculpture by Edgar Degas. The authors were unable to present results but exemplified the challenges of analyzing such a complex, multimodal data set. With regard to paintings, Walker et al. (60) conducted a remarkable MET study in the Van Gogh Museum in Amsterdam. However, the researchers used fiducial markers displayed around the frames (altering the museum display), were continuously present, and had the participants follow fixed positions and viewing times. In the aforementioned study on installation art, in which Pelowski et al. (52) recorded eye movements from twenty-four participants, these were able look at the exhibition without a pre-given path or time restrictions. Nonetheless, due to the small sample size and the fact that all participants were psychology students, they concluded that “this topic remains a largely under-explored avenue for future empirical work” (52, p. 20).

In addressing the current limitations of MET studies in museums, we need to strive for the most authentic study conditions and larger sample sizes in order to reach ecologically valid and generalizable findings. This was the aim and case of our study “Belvedere Before and After.” We seized the opportunity provided by the museum’s redisplay of its permanent collection—something that occurs only once every two decades or so—to analyze visitors’ approaches to the same artworks in two different display constellations. The study was conducted as a collaboration between the Laboratory for Cognitive Research in Art History (CReA) at the Department of Art History of the University of Vienna, the Perception Engineering Group at the Department of Computer Engineering of the University of Tübingen, the Research Focus *Empirical Visual Aesthetics* (EVAlab) at the Department of Psychology of the University Vienna, and the Austrian Gallery Belvedere. This allowed us to combine the expertise of art historians, museologists, psychologists, and computer scientists. Overall, the study benefited from the comparison of two realistic display conditions (not artificially set up for research purposes), implemented an unconstrained study design (working with regular museum visitors who experienced the exhibition in natural groups and according to their own preferences), and managed to collect a large data sample (259 participants in total).

However, applying MET in a large-scale field study poses some technical challenges and comes with restrictions in data analysis. While we were able to demonstrate that MET technology is advanced enough to record data in such an unconstrained study design (61), it also became evident that reported MET data still lacks accuracy (e.g. with respect to eye movements within single artworks) and is not yet able to provide automatically reported semantic mappings (e.g. with respect to eye movements between artworks). Since the output of the eye tracker are gaze positions in the scene camera’s video rather than coordinates of the museum space, we opted for the manual coding of the videos with respect to our objects of interest, i.e. mainly artworks and labels.

This paper presents the specifics of our field study and discusses the results of initial data analysis – focusing on the parameter of viewing time and a sub-sample of 100 participants.

## Methods

In accordance with the necessities of a field study on art perception embedded in an authentic museum experience, our research approach was exploratory and guided by an open research question: How does the display influence the way people see and experience art in a museum? This question relates to the institutional, curatorial, and spatial frameworks that provide a script for visitors to view different artworks and stimulate their subjective meaning-making processes. Conducting a study in ecologically valid conditions means that it is neither possible nor desirable to isolate and control single variables such as the number of artworks or people present. We therefore also did not formulate detailed hypotheses about the influence of the display on eye movements. Instead, we have tried to discern specific effects of the museum’s rearrangement by applying and combining diverse analytical tools, a mixed-methods approach combining quantitative and qualitative methods: MET was complemented with self-reported visual and verbal data (subjective mapping) as well as detailed contextual information on visitors’ backgrounds (questionnaire).

### Procedure

The data collection took place January 22–28, 2018, for the first part “Belvedere Before” (hereafter BB), and from January 28 to February 3, 2019, for the second part “Belvedere After” (hereafter BA); in both cases from Monday to Sunday so as to cover an entire museum week with comparable seasonal and day-specific visitor profiles. Both parts of the study followed identical procedures (see video https://crea.univie.ac.at/projects/belvedere-before-and-after): First, visitors were informed about the study and invited to participate. Those who accepted signed a consent form and were equipped with one of the four available eye tracking devices consisting of a Pupil Labs Headset (Pupil Core) with two adjustable eye cameras and one scene camera capturing the participant’s field of vision. The headset was connected to a Microsoft Surface Pro 4 tablet PC worn in a light backpack (< 1 kg). The eye tracking data was recorded with the software EyeRecToo (62), and calibration was conducted as proposed in CalibMe (63). This eye tracking software offers several distinct methods for implementing functionality in the eye-tracking pipeline: We configured it with PuRe for pupil detection (64); with PuReST for pupil tracking (65); and with Grip for gaze estimation (66). After visiting an acclimatization room and the three rooms included in our study (see Figure 1), each participant took part in the subjective mapping task (see Figure 2) and answered the questionnaire. There was no monetary compensation, but participants received a small (unannounced) gift from the museum’s shop.

**Figure 1.**
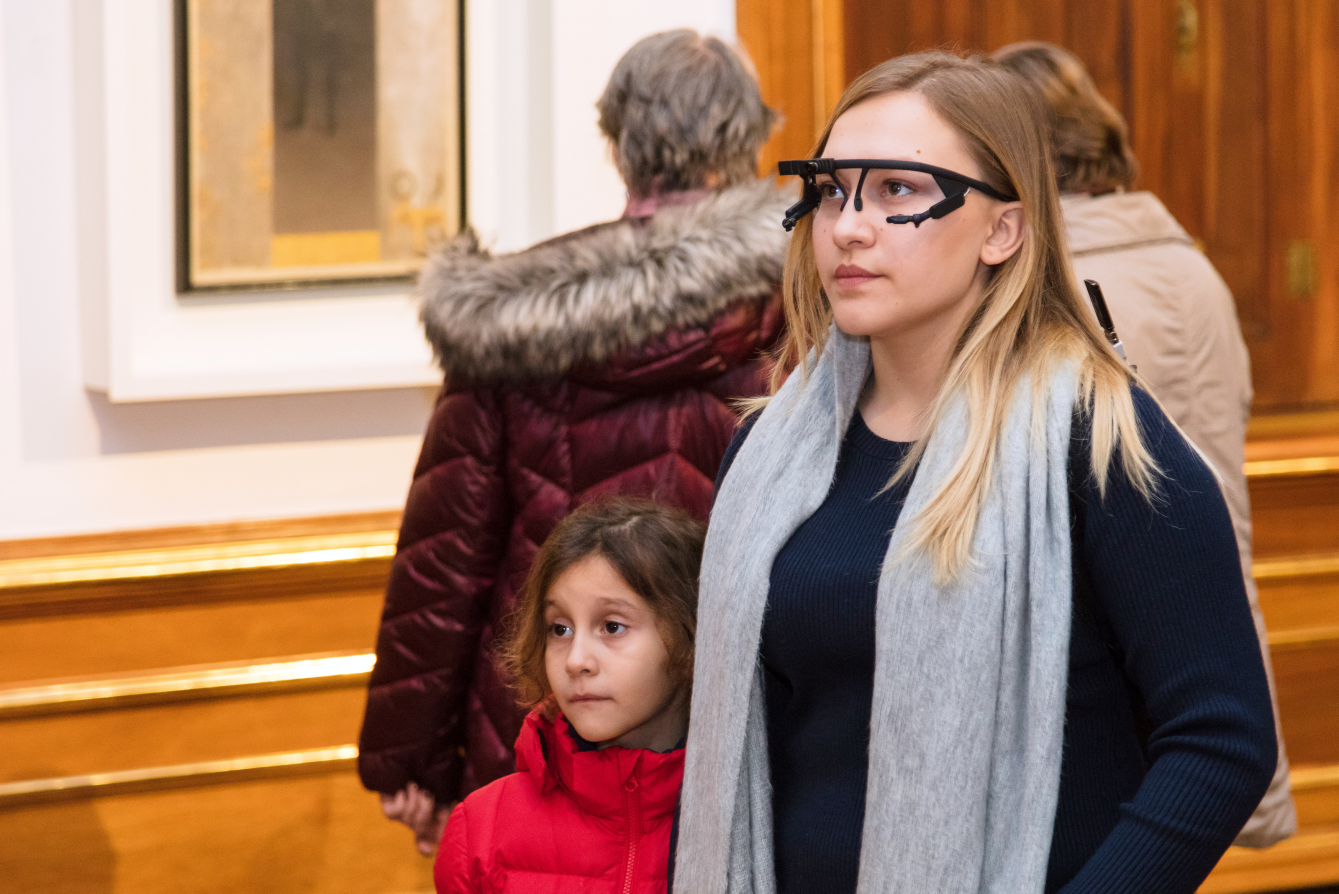
Individual exhibition visit with MET equipment. © Department of Art History, University of Vienna

**Figure 2.**
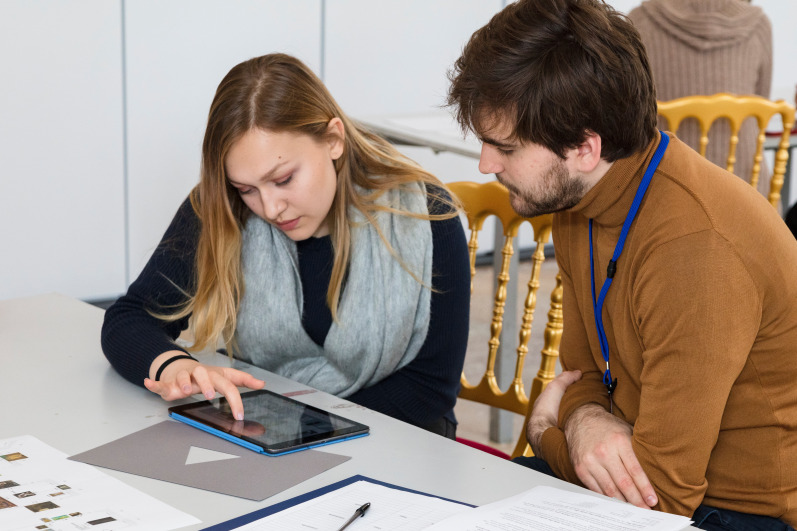
Subjective mapping following the exhibition visit. © Department of Art History, University of Vienna

### Mobile eye tracking

MET has already been applied in museum studies (see above) and proven to be an insightful method due to the richness of the data gained, which also enables statistical analysis (67, 68, 69). However, one of the main challenges of employing MET is extracting semantic meaning from the raw gaze data in relation to the stimuli (e.g., a painting on the wall of the museum), as these stimuli are consistently viewed from different perspectives. The eye movement data regarding the objects looked at (e.g. a specific artwork or label) is not a direct output of the recording software but a video of the scene camera with an estimated gaze point relative to the video frame. Moreover, regular fast-paced head movements (leading to motion blur in the captured images), the low dynamic range of the eye tracker’s field camera, and frequent partial occlusion of artworks due to the presence of other visitors make the automatic labeling a challenging task. Given these technical limitations, we favored manual annotations for semantic mapping in this project—a labor intensive but more robust approach.

In our manual annotations, we assigned a code to each of the elements considered relevant in the exhibition space by means of the self-developed application Eye Movement Coder (current version 2.5). The particular object of interest could be selected from a predefined list, including all of the artworks, texts, people, media, and markers. As for the rooms before their rearrangement, fiducial markers were used to identify three artworks for potential automatic image recognition in later data analysis. The annotation process consisted of establishing and “stamping” the start and end time of continuous periods of attention on a specific object of interest. Each video was viewed and annotated by one coder, edited by a second, and finally checked by a supervisor. Four videos were randomly selected and annotated a second time by yet another person who was unaware of the first annotation. This facilitated a strong agreement between the two coders (Cohen’s Kappa = > 0.84). Taking advantage of this manual annotation process, we employed a functional definition of fixation (rather than a computational one) as judged by the human coders (70). Additionally, multiple looks on objects of interests such as artworks and labels were summed up. Thus, our data analysis employed the parameter of viewing time per object of interest instead of algorithm-dependent metrics such as number of fixations or average fixation duration.

### Subjective Mapping

While MET tracked the fast and jumping gaze between objects, people, and space, the subjective mapping allowed us to relate viewing behavior to individual meaning-making processes through visitors’ a posteriori reports on their exhibition visits. Historically, such mapping techniques refer to mental maps as cognitive representations of spatial experience (71) as well as to focused interviews using stimuli to elicit conversation (72). Mapping tools have also been developed for museum and visitor studies (73, 74, 75, 76). For the subjective mapping applied in our study, we asked participants to mark the areas they remembered the strongest by drawing on an illustrated floor plan on a tablet. This task formed the basis for the simultaneous open interview wherein visitors reconstructed their exhibition experience, including reactions to specific works they could easily refer to on the map.

The process by which we prepared the subjective mapping data consisted of transcribing the interviews and annotating the artwork references. We named the artworks in brackets when a visitor declared, for instance, that he remembered “the paintings of Van Gogh [Auvers a2_10] and Munch [Männer a2_11], because I know the painter” (ba-s008). With the help of NVivo, a qualitative data analysis program, we categorized the interview statements according to the same coding grid as applied to the MET videos, differentiating between references to artworks, texts, and media. Additional codes, which pre-categorized topics and text segments for later content analysis, referred to the curatorial design of the three rooms as well as to museum and art interest in general.

### Questionnaire

In order to broadly frame visitors’ background and museum experience, we also used a comprehensive questionnaire (see Figure A1 in the Appendix) with six parts related to 1) artworks seen beforehand or personal highlights; 2) the exhibition experience in relation to display issues; 3) personal art interest, evaluated through the art interest scale of the Vienna Art Interest and Art Knowledge questionnaire (77); 4) specifics of the museum visit, e.g. alone, in a group or as tourists; 5) feedback on the use of MET, focusing on usability; and 6) socio-demographic background. The questionnaire was implemented with the survey program Qualtrics and displayed on an iPad handed to participants. Answers were mainly given on 7-point Likert scales. Statistical data analysis was conducted in R.

### Participants

Our total sample consisted of 259 visitors, of whom 109 participated in BB and 150 in BA, with a mean age of 33.54 ( *SD* = 14.12); mean age was comparable across both groups (BB: *M* = 34.9, *SD =* 14.7; BA: *M* = 32.6, *SD* = 13.7). In total, 112 participants were male (BB: 46; BA: 66), 146 were female (BB: 63; BA: 83), and 1 person (in the BA group) did not have a binary gender identity. Most of our participants visited the museum in a group context (Total: *N* = 198; BB: *N* = 77; BA: *N* = 121), with friends and families (Total: *N* = 159; BB: *N* = 61; BA: *N* = 98) as well as pairs (*N* = 104; BB: 33; BA: 71) as the most common group constellation. The majority of our participants were tourists (Total: *N* = 201; BB: *N* = 81; BA: N = 120), which was reflected in English being the predominant language of participation (Total: *N* = 202; BB: *N* = 75; BA: *N* = 127; all others German) and most participants visiting the Belvedere for the first time (Total: *N* = 224; BB: *N* = 85; BA: *N* = 139). Participant characteristics were thus comparable across our two groups.

For this paper, we have limited the analysis to a total of 100 participants due to the time-consuming nature of the manual annotation process. The selection of these 50-50 data sets out of the 109 in BB and 150 in BA was based on the data quality (e.g., slippage, 78) and on the subset’s representativeness of the whole data set with regard to gender (42 men, 58 women), age (mean 33.32, *SD* = 13.45), and visiting specifics (76 tourists, 24 non-tourists). Since the results showed an influence of the level of participants’ art interest on viewing times, we checked if that interest differed between the BB and BA participants. This proved not to be the case (BB: *M* = 49.59, *SD* = 13.51; BA: *M* = 48.97, *SD* = 12.66; t(257) = -0.38, *p* = .070), i.e. the two subsets had similar means of art interest and were thus also comparable in this regard.

### Materials

The stimuli of our study were original artworks, i.e., paintings and sculptures in an authentic museum context: the Baroque palace’s venue, also known as the Upper Belvedere, which showcases the permanent collection of the Austrian Gallery Belvedere (see online database https://www.belvedere.at/en/collection). While the old display had been the subject of minor adaptations over the past two decades, the new display aims to provide a continuous course through 700 years of Austrian art history. The redisplay of the permanent collection aimed to offer “fresh approaches to these masterpieces” including “innovative thematic rooms, interspersing the chronological hanging through the periods of art and sparking a multi-layered dialogue between the classics of art history and contemporary artists” (79). Since Gustav Klimt’s paintings are the best-known works of the museum, we chose three rooms with his artworks and those by artists of his time for our study.

These rooms underwent three major display changes during the museum’s rearrangement: First, where the old display used a variety of wall colors, the new one relies on uniformly white walls. This goes hand in hand with specific curatorial styles: While the bluish-tinted wall colors of Rooms 1 and 2 of the BB constellation reference either the salon presentation style of the 19^th^ century or postmodern wall color experiments, the new BA display relates to the dominant presentation mode of the 20^th^ century, the “White Cube,” which highlights single objects in a reduced setting. The white background is also used for the presentation of the core Klimt piece “The Kiss,” which is now displayed on a free-standing wall at the end of the room sequence. Earlier, “The Kiss” had been shown in Room 3, embedded in a black side wall, with his other works presented on white walls, aside from the red and black background for the small-format painting “Girlfriends (Water Serpents I).”

Second, the art historical narration in the single exhibition rooms has changed drastically. This includes a new exhibition course, with most of the pieces of our set now in the East wing instead of the West wing of the building. In BB the course of the rooms synthesized a narration that led from works by Hans Makart, the most successful painter in Vienna before Klimt (in our acclimatization room), to the presentation of the Viennese Secession (in Rooms 1 and 2) to a rich compilation of Klimt’s works including the most famous, “The Kiss” and “Judith,” at opposite far end walls (in the large Room 3). In BA, Klimt’s paintings are no longer presented together in one large room, but distributed according to chronology and motifs; moreover, they are now matched with works by fellow contemporary artists. Besides curatorial reasons, this change was intended to affect the stream of visitors eager to see Klimt’s works. The crowded situation of the single Klimt room in the BB setting was equalized by spreading his works over several rooms. The new BA display includes a variety of smaller thematic units, showing more works by more artists: Vincent Van Gogh’s “Plain of Auvers,” Giovanni Segantini’s “The Evil Mothers,” and Max Klinger’s “Crouching Woman” have lost the central position they held in BB. They are now part of a long row of works by artists of the Viennese Secession. The BA display also shows more sculptures (seven instead of three), mainly positioning them close to the walls in line with the paintings or alongside the room passages.

Third, another major change was the introduction of interpretive labels (80, p. 19–29), which were completely missing before; these take the forms of introductory room texts and captions for some artworks. The new display thus represents a shift from the simple identificatory label as the only textual information source in BB to a three-layered information structure in BA. In this way, texts on white stelae, as a first layer, offer short introductions to each room (“Vienna around 1900: All the World’s a Stage” in Room 1; “The Secession” in Room 2; “Gustav Klimt” in Room 3). Additionally, the stelae include a sketch and a brief text concerning the historical decoration and function of each room. A second layer (consistent with that of BB) is each artwork’s identification label (i.e., artist, place, year of birth and death, title and year of the artwork, technique, dimensions, and – where relevant – audio guide or sign language number; a new addition is information regarding the artwork’s provenance). As a third layer, newly added captions on six out of thirty-five artworks (or four out of the thirteen presented in both BB and BA) provide information on the artwork’s context, theme, and style. While the bilingual language structure with German and English has remained, the visual appearance of the labels has changed from the former white or dark grey letters pasted on the walls to cardboard labels with grey text against white backgrounds (and different shades of grey for language differentiation).

In summary, our material consisted of twenty-six artworks in BB (including three sculptures) and thirty-five artworks in BA (including seven sculptures) (see Figure 1, Table A1, and A2 in the Appendix). Thirteen artworks were shown in both display conditions, with four of them receiving new interpretive labels in the form of captions in BA.

**Figure 3.**
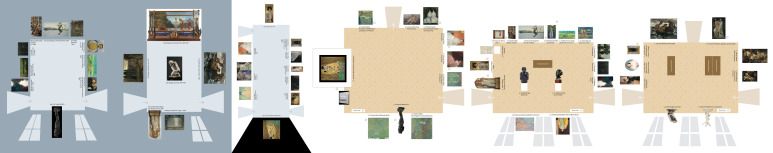
Map with 26 artworks in BB and map with 35 artworks in BA

## Results

As outlined above, our study was conducted as field research in an authentic exhibition setting, where we investigated visitor behavior at two different points in time. The results of this first data analysis report the changes and consistencies that we encountered when comparatively analyzing the BB and BA data. Starting with a MET usability note, results on the general distribution of attention are followed by those on time spent viewing artworks, distinguishing between painting and sculpture. Further, we report findings on specific artworks in the focus of attention and the combined activity of looking at art and reading text, including the influence of personal characteristics.

### Usability

The usability of the MET equipment was assessed in our questionnaire. An evaluation of the results for the BB data has already been published in Santini, Brinkmann et al. (2018). We ran t-tests for both data sets (*n* = 259) and found no significant differences in usability between BB and BA ( *p* < .05). Questions, to be answered on 7-point Likert scales (1 = not all, 7 = very much), included one about the influence of the equipment on art experience: “My art perception has changed because of the eye-tracking equipment”. In general, people indicated that their experience was not influenced by wearing the eye tracking glasses (BB: *M* = 2.35, *SD* = 1.64; BA: *M* = 2.54, *SD* = 1.71). This supports the idea that MET is a valid and reliable technology for registering the museum gaze and for decoding the display effect.

### General distribution of attention

The annotated MET videos from the 50:50 participants resulted in a total time of 34,869 sec (9 h 41 min 9 sec) in the BB and 54,315 sec (15 h 5 min 5 sec) in the BA constellation, counting the time from the first to the last video annotation in the three rooms. This means that the average dwell time per visitor increased from 11:37 min to 18:06 min. However, the dimensions of the rooms had also changed (see Materials above), with an increase in size from 305.20 m^2^ to 387.70 m^2^, as did the number of artworks displayed, from twenty-six to thirty-five. Nevertheless, we observed an increased awareness per square meter as visitors looked at 26.26 m^2^ per minute in BB and at 21.41 m^2^ in BA on average. Comparison of the distribution of attention in BB and BA (see Table 1) shows a temporal dominance of looking at art in the BB setting, where almost no text and only very plain label information was offered. There, participants spent 20,068 sec or 57.27% of their total measured time looking at artworks and only 2,472 sec or 7.05% on reading text. This ratio changed in the BA setting, where additional interpretive labels resulted in participants spending 26,125 sec or 48.10% of their total time looking at artworks and 11,443 sec or 21.07% reading text. While participants did not look at artworks for shorter periods of time in general (see Results below), the relation between looking at art and looking at text changed drastically from a rough 8:1 to 2:1 ratio. However, the ratios of spending time with media (audio guides, phones, photo cameras), looking at people (companions or other persons present), and other elements (non-annotated time looking somewhere else, e.g., at windows and floors) remained nearly the same. Although the participants of our study spent only 174 seconds or 0.50% with fiducial markers that were applied to three out of twenty-six artworks in the BB constellation (to enable an automatic image recognition potentially applied in later data analysis), we decided not to use markers in the BA constellation, as the MET videos proved a major distraction of attention towards the markers placed around the paintings in the authentic exhibition context.

**Table 1:** Cumulated viewing times in seconds and percentages in BB and BA

	Belvedere Before		Belvedere After	
	sec	%	sec	%
Artwork	20,068	57.27	26,125	48.10
Text	2,472	7.05	11,443	21.07
Media	1,888	5.39	1,932	3.56
People	2,797	7.98	3,233	5.95
Marker	174	0.50	/	/
Other	7,644	21.81	11,582	21.32
Total	34,869	100	54,315	100

### Time spent viewing artworks

Regarding the average time visitors spent looking at artworks (see Tables 2 and 3), we note that the viewing time per artwork differs greatly: Some received very little and others considerable attention. In BB, the viewing times for the twenty-six artworks ranged from a mean viewing time of 56.94 sec (*SD =* 38.57) for “The Kiss” by Gustav Klimt to 4.91 sec ( *SD* = 5.94) for “Calm Water” by Fernand Khnopff. In BA, there was an even broader range of viewing times between the thirty-five artworks. The longest mean viewing time with 57.92 sec ( *SD* = 48.54) was, again, observed for the “The Kiss” by Klimt and the shortest mean viewing time of 2.10 sec ( *SD =* 4.73 sec) for “Gustav Mahler” by Auguste Rodin.

**Table 2:** Viewing times in seconds for the 26 artworks and their labels in BB

	Belvedere Before	Artwork		Label	
N°	Abbreviated titles	Mean (*SD*)	Median	Mean (*SD*)	Median
1	Kiss	56.94 (*38.57*)	47.00	1.70 (*3.33*)	0.00
2	Judgement	38.87 (*38.16*)	29.22	2.60 (*4.29*)	0.00
3	Bride	37.37 (*29.26*)	29.42	2.53 (*3.02*)	1.43
4	Judith	24.44 (*21.68*)	19.10	1.69 (*2.04*)	0.77
5	Fritza Riedler	23.86 (*16.16*)	19.12	2.20 (*3.13*)	1.15
6	Girlfriends	21.19 (*20.11*)	15.75	2.93 (*3.73*)	2.07
7	Evil Mothers	21.03 (*18.66*)	15.58	2.41 (*3.01*)	1.43
8	Pax	20.50 (*18.22*)	14.85	1.50 (*2.89*)	0.00
9	Sea Idyll	15.66 (*12.81*)	12.45	1.93 (*2.44*)	1.45
10	Josef Lewinsky	13.26 (*11.45*)	11.57	3.05 (*2.82*)	2.47
11	Plain Auvers	13.15 (*12.69*)	11.40	2.77 (*3.67*)	1.63
12	Amalie Zuckerkandl	11.52 (*9.76*)	9.77	2.55 (*3.03*)	1.67
13	Largo	10.72 (*13.17*)	7.65	1.77 (*2.32*)	0.83
14	Lady White	10.17 (*8.24*)	7.83	1.18 (*1.41*)	0.87
15	Allegory Music	9.06 (*7.37*)	7.95	2.15 (*2.64*)	1.53
16	Early Spring	8.70 (*8.78*)	5.23	2.47 (*3.26*)	1.32
17	Painter Physician	8.28 (*7.58*)	5.58	3.86 (*4.88*)	2.12
18	Lady Fireplace	8.23 (*7.62*)	6.33	1.59 (*1.98*)	1.05
19	Pond	7.34 (*5.81*)	5.62	2.00 (*2.94*)	1.40
20	Dachstein	6.66 (*8.42*)	4.55	1.03 (*1.68*)	0.00
21	Nymph	6.65 (*7.87*)	4.13	0.70 (*2.01*)	0.00
22	Crouching Woman	6.27 (*12.69*)	1.57	0.14 (*0.60*)	0.00
23	Self-Portrait	6.10 (*7.66*)	2.97	1.75 (*1.91*)	1.22
24	Twilight	5.38 (*6.05*)	3.30	1.67 (*2.20*)	0.87
25	Eve	5.09 (*6.78*)	2.63	0.00 (*0.00*)	0.00
26	Calm Water	4.91 (*5.94*)	2.73	1.28 (*1.82*)	0.50

**Table 3:** Viewing times in seconds for the 35 artworks and their labels in BA

	Belvedere After	Artwork		Label	
N°	Abbreviated titles	Mean (*SD*)	Median	Mean (*SD*)	Median
1	Kiss	57.92 (*48.54*)	42.58	14.09 (*21.61*)	1.80
2	Evil Mothers	30.58 (*33.39*)	22.32	4.75 (*5.01*)	2.75
3	Judith	30.30 (*31.02*)	21.67	16.51 (*17.51*)	8.27
4	Fritza Riedler	27.60 (*27.80*)	19.53	3.21 (*3.65*)	2.18
5	Sonja Knips	21.39 (*22.15*)	13.45	11.05 (*11.27*)	9.88
6	Sea Idyll	21.31 (*16.60*)	16.47	3.83 (*5.71*)	2.08
7	Forester’s House	20.12 (*24.79*)	10.38	4.20 (*4.80*)	2.55
8	Lady Black	19.88 (*17.65*)	13.73	4.11 (*5.00*)	2.90
9	Antique Sacrifice	18.61 (*19.25*)	13.72	3.68 (*4.33*)	2.90
10	Josef Lewinsky	18.15 (*25.05*)	9.67	14.74 (*18.27*)	6.60
11	Plain Auvers	15.84 (*18.20*)	9.93	13.50 (*16.33*)	6.07
12	Orpheus Eurydice	14.69 (*19.25*)	7.87	3.64 (*5.65*)	2.08
13	Cottage Garden	14.40 (*14.83*)	8.25	2.08 (*2.41*)	1.13
14	Donaulände Summer	13.85 (*16.37*)	10.57	3.60 (*3.72*)	2.60
15	Seashore	13.83 (*14.24*)	10.90	4.42 (*5.82*)	2.22
16	Flowering Poppies	12.98 (*16.32*)	7.12	1.84 (*2.79*)	0.53
17	Emotion	12.83 (*14.52*)	6.75	3.16 (*3.39*)	2.33
18	Schloss Kammer	12.82 (*12.34*)	8.87	4.63 (*5.43*)	3.60
19	Sisters Fey	12.78 (*16.43*)	3.37	8.00 (*10.72*)	2.07
20	Therese Bloch-Bauer	12.23 (*15.37*)	8.45	3.18 (*3.57*)	2.07
21	Marie Kerner	12.18 (*8.94*)	9.65	4.39 (*4.58*)	3.05
22	Dante Vergil	12.10 (*13.23*)	9.03	4.17 (*5.29*)	2.48
23	Nymph	11.53 (*18.16*)	6.38	3.35 (*4.17*)	2.27
24	Lost	11.53 (*14.93*)	7.08	3.22 (*3.58*)	2.60
25	Pond	11.01 (*21.71*)	5.90	3.18 (*3.85*)	1.37
26	Visitation	10.75 (*12.87*)	5.05	3.06 (*4.46*)	1.78
27	Early Spring	9.47 (*9.40*)	6.02	2.67 (*2.61*)	1.97
28	Twilight	8.86 (*10.89*)	4.12	2.64 (*3.23*)	1.48
29	Cupid Psyche	8.36 (*10.22*)	5.98	2.09 (*4.66*)	0.00
30	Eve	7.40 (*13.73*)	2.30	1.48 (*2.02*)	0.08
31	White Poplars	5.67 (*5.71*)	3.93	2.59 (*3.08*)	1.33
32	Crouching Woman	4.19 (*6.85*)	2.38	1.77 (*3.05*)	0.43
33	Woman Bathing	3.12 (*3.98*)	1.48	1.47 (*2.54*)	0.82
34	Beethoven	2.13 (*5.03*)	0.53	1.03 (*2.93*)	0.00
35	Gustav Mahler	2.10 (*4.73*)	0.52	0.68 (*1.47*)	0.00

Comparison of attention per artwork in the three rooms from BB (with twenty-six artworks) and BA (thirty-five artworks) reveals only a small decline of the mean viewing time from 15.44 sec ( *SD* = 21.18) to 14.93 sec (*SD =* 21.25). Interestingly, this decline is not even present for the art form of painting; here, the mean viewing time slightly rises from 16.67 sec (*SD =* 21.97) to 17.27 sec (*SD =* 22.55). Regarding the art form of sculpture, we see a small decline from the mean viewing time of 6.00 sec (*SD =* 9.43) to 5.55 sec (*SD =* 10.69) in the BA presentation, where seven instead of three sculptures were presented in the three rooms (see Table 4). This means that, although a higher number of paintings and sculptures was presented in the BA constellation, the average attention span per artwork only decreased slightly.

**Table 4:** Viewing times in seconds per painting, per sculpture and per artwork in BB and BA

	Belvedere Before		Belvedere After	
	Mean (*SD*)	Median	Mean (*SD*)	Median
Sculpture	6.00 (*9.43*)	2.77	5.55 (*10.69*)	1.72
Painting	16.67 *(21.97*)	9.97	17.27 (*22.55*)	9.80
Artwork	15.44 *(21.18*)	8.58	14.93 (*21.25*)	8.07

### Painting versus sculpture

Consequently, the major difference we found in comparing the mean viewing time per artwork in BB and BA is not related to the difference of display but of art form, i.e., painting versus sculpture. This observation regarding a preference in art forms is also supported by the specific viewing time ranks detected in BB and BA (see Tables 2 and 3). In BB, the three sculptures “Half-figure of a Nymph (‘Vivien’)” by Fernand Khnopff, “Eve” by Auguste Rodin, and “Crouching Woman” by Max Klinger occupy the lowest ranks, i.e. 21, 22, and 25 (out of twenty-six). In BA, the seven sculptures (including the three just mentioned, plus “Cupid and Psyche” by Theodor Friedl, “Woman Bathing” by George Minne, “Gustav Mahler” by Auguste Rodin, and “Beethoven” by Max Klinger) also occupy the lowest ranks, i.e. 23, 29, 30, 32, 33, 34, and 35 (out of thirty-five). Thus, the MET data clearly shows less interest in sculpture (or even a neglect of it) than in painting. This correlates with findings from the questionnaire and subjective mapping. Visitors indicating in the questionnaire that they were mainly interested in painting proved predictive of a longer viewing time for paintings (*R2* =.03, *F*(1,98) = 2.66, *p* = .11, *B* = 43.75). Similarly, visitors indicating that they were mainly interested in sculpture proved predictive of a longer viewing time for sculpture (*R2* =.03, *F*(1,98) = 2.71 *p* = .10, *B* = 4.39). Both correlations were assessed with a linear regression; however, neither of these analyses was significant and the explained variance was very low. Moreover, the subjective mappings indicate a preference for painting over sculpture when visitors said they “didn’t look much at the statues” (ba-s009), “don’t remember seeing the statues” (ba-s012), or found them “not very interesting” (ba-s038), especially in BA. However, this is not a universal rule, as some visitors also emphasized their special interest in sculptural works.

Going beyond this general, weakened attention pattern regarding the art form of sculpture, we can discern a display effect among the three sculptures exhibited in both constellations (see Tables 2 and 3). While the viewing time of “Eve” slightly increased (BB: *M* = 5.09 sec, *SD* = 6.78; BA: *M* = 7.40 sec, *SD* = 13.73), “Crouching Woman” attracted less attention (BB: *M* = 6.27 sec, *SD* = 12.69; BA: M = 4.19 sec, *SD* = 6.85), and “Half-figure of a Nymph (‘Vivien’)” clearly gained in attention (BB: *M* = 6.65 sec, *SD* = 7.87; BA: *M* = 11.53 sec, *SD* = 18.16). In the case of “Eve,” we encountered the interesting case that none of the 50 BB participants looked at the label (being placed far away from the work); thus, no one could easily attribute the work to Rodin. In the case of “Crouching Woman” by Klinger—the only artwork among the thirteen presented in both conditions that was looked at for a shorter period of time after the museum’s rearrangement—we can explain

the loss of attention with its new positioning. While in BB, the sculpture was presented in the middle of the room in line with the artist’s demand for viewers to walk around and see it from multiple perspectives (81, 82), the BA presentation places the work close to a wall and in front of a mirror. The “Half-figure of a Nymph (‘Vivien’)”, in contrast, gains in attention by being newly positioned next to the increasingly popular “Judith” by Klimt (see Results below). This new position contributes spatially, as the sculpture is positioned directly in the default line of sight when entering the second room, as well as in terms of content, as visitors in their meaning-making confront and compare two artworks depicting seductive women.

### Specific artworks in the focus of attention

Comparing the viewing times for the thirteen artworks that were both presented in BB and BA (see Figure 4 as well as Table A3 and A4 in Appendix), we detect a clear rise of attention after the rearrangement of the rooms. The mean viewing time per artwork increased from 15.98 sec (*SD* = 21.08) to 19.55 sec ( *SD* = 27.77). These results show that the new BA presentation generally stimulates visitors to spend more time with these artworks, with a mean attention shift of 22.34% per work. However, the ranking

**Figure 4.**
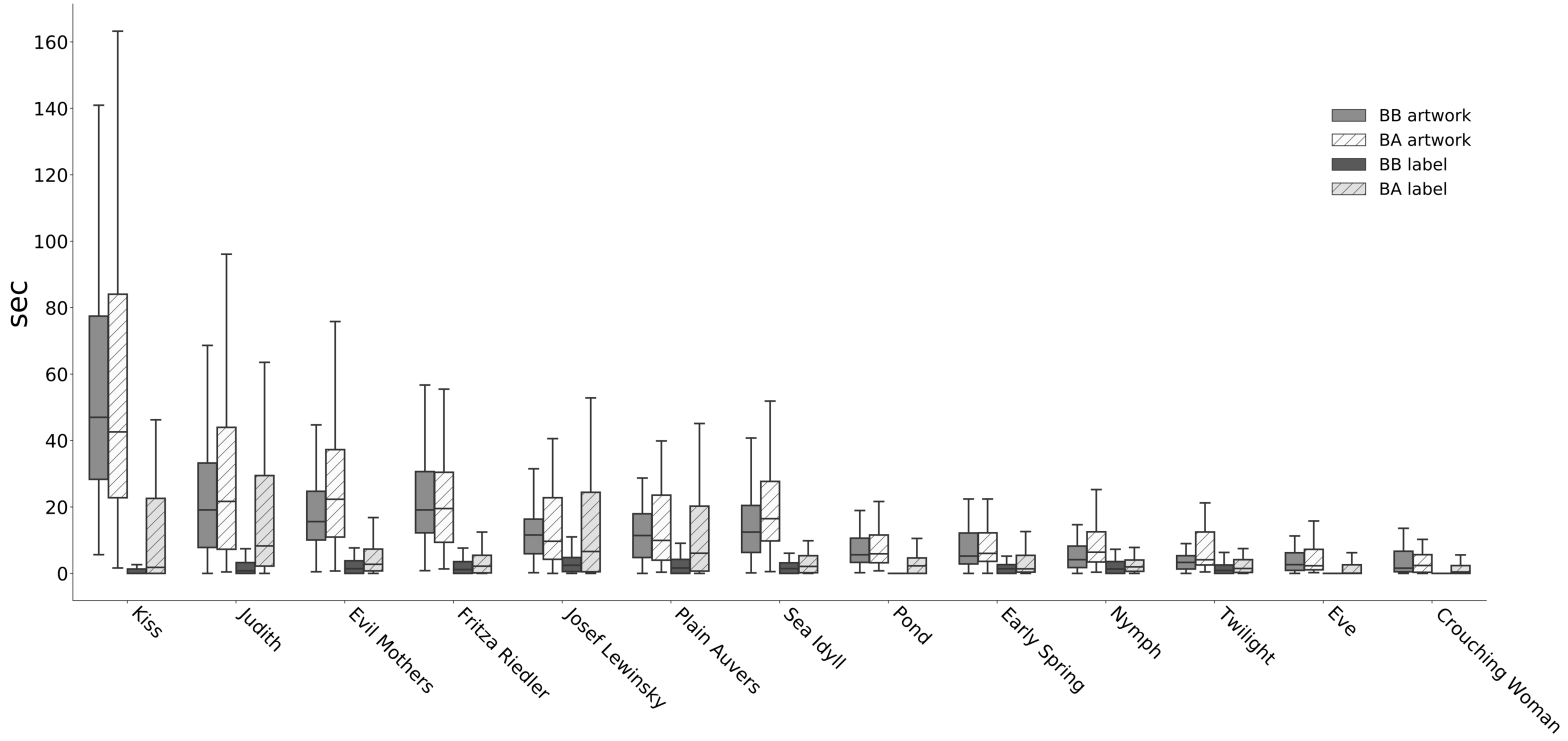
Average viewing times in seconds for the 13 artworks in BB and BA

according to viewing times shows a strong consistency between the higher and lower ranks of these thirteen artworks in BB and BA. Regardless of their display, certain artworks remain in the focus (see ranks 1–7), while others consistently receive less attention time (see ranks 8–13). Among the artworks in the focus of attention, there is of course “The Kiss,” (see Figures 5 and 6) as participants often stated in the subjective mappings when they recalled their exhibition visit. This is not surprising, as “The Kiss” is the most prominent artwork of the museum and one that most visitors were already familiar with, having seen it before either in the original or as a reproduction (BB 77.98%, BA: 74.67%). In addition, works by widely known artists such as Klimt, van Gogh or Munch led to a heightened awareness, as visitors felt they “had to stop because it was from a famous painter” (ba-s075) or “of course […] had to pay attention” (bb-s001) to these works.

**Figure 5.**
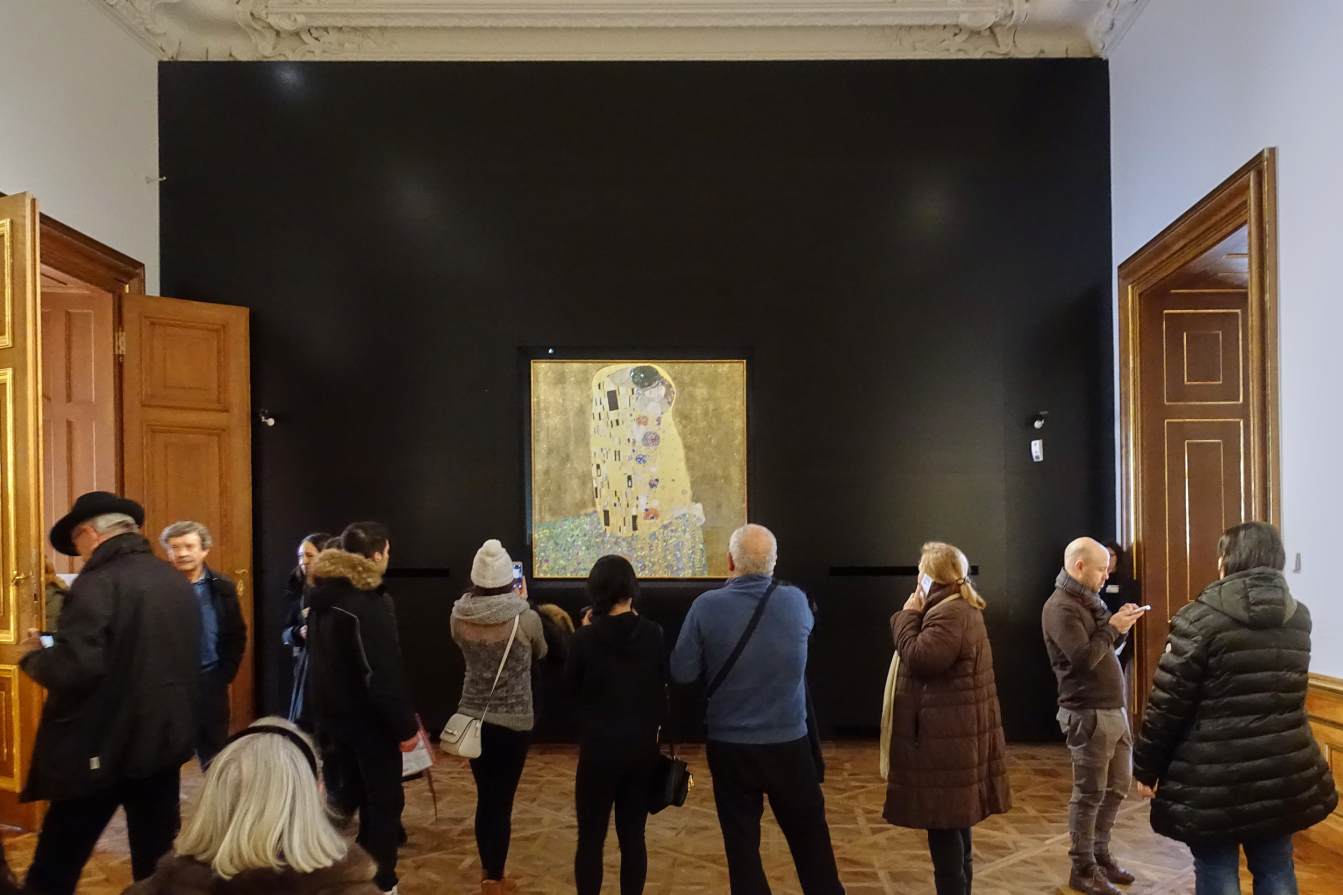
Installation shot with “The Kiss” in BB. © Department of Art History, University of Vienna

**Figure 6.**
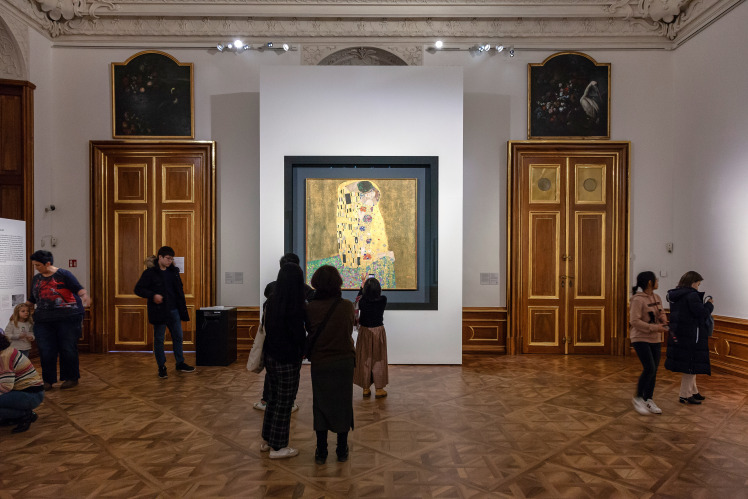
Installation shot with “The Kiss” in BA. © Department of Art History, University of Vienna

In comparison to other artworks in the higher ranks, where three out of seven change their ranks, “The Kiss” remains the clear number one in terms of attention time. More specifically, the artwork holds an extremely high mean viewing time of almost one minute in BB ( *M* = 56.94 sec, *SD* = 38.57) as well as in BA (*M* = 57.92 sec, *SD* = 48.54). Accordingly, “The Kiss” also received the highest number of looks (BB: *M* = 27.62, *SD* = 25.76; BA: *M* = 28.02, *SD* = 23.25) compared to the average number of looks per artwork (BB: *M* = 7.58, *SD* = 8.69; BA: *M* = 9.08, *SD* = 10.28). The repeated returns to the work can be ascribed to the fact that the exhibition room tends to be crowded (which leads visitors to look at other visitors, or at other artworks, while waiting to look at “The Kiss”) and the need to evaluate the famous artwork for oneself in several steps. Due to this viewing context, “The Kiss” consistently required time to consume—although with differing conclusions. Many of the statements from the subjective mapping make it clear that the work was remembered for being famous. A small group of responses further demonstrates that “The Kiss” is a piece that polarizes: While for some visitors the artwork was the principal reason to come to the museum, with enthusiastic reactions such as “it made me feel loved, because it just depicts love” (bb-s004), other visitors found the piece “famous but […] not interesting at all” (bb-s006) or simply too “commercial” (ba-s126).

**Figure 7.**
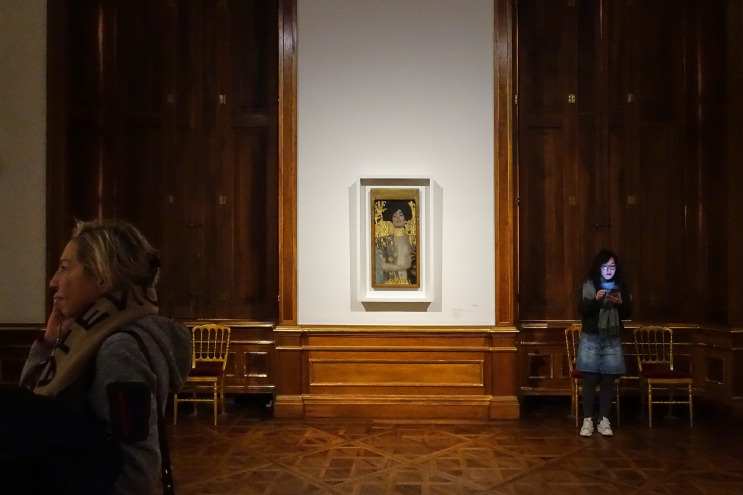
Installation shot with “Judith” in BB. © Department of Art History, University of Vienna

**Figure 8.**
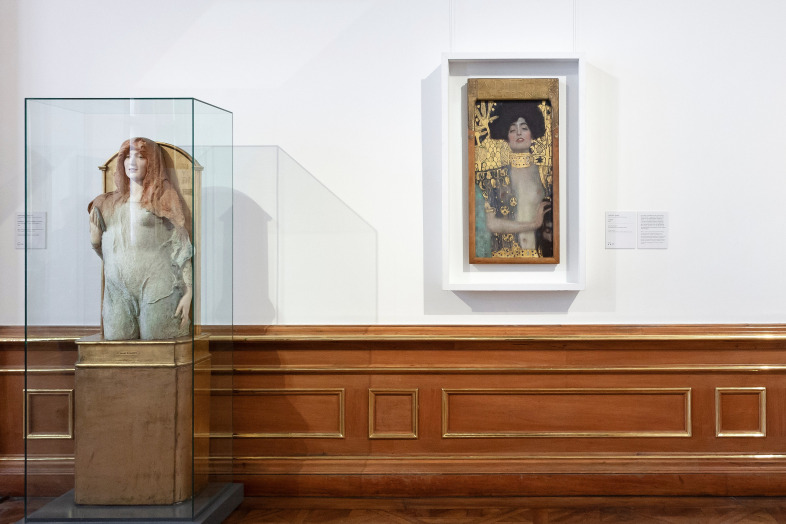
Installation shot with “Nymph” and “Judith” in BA. © Department of Art History, University of Vienna

**Figure 9.**
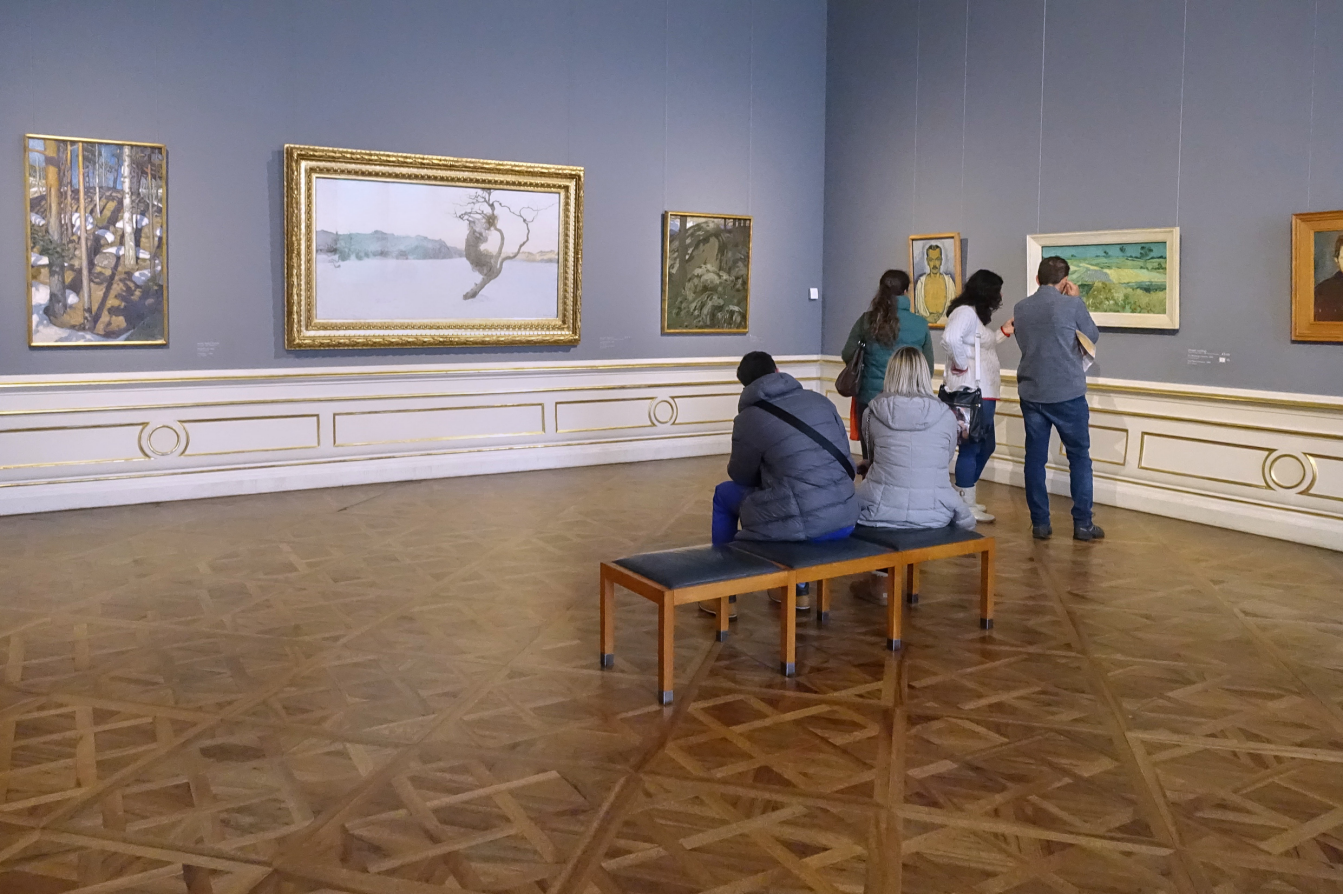
Installation shot with “Evil Mothers” (center) in BB. © Department of Art History, University of Vienna

**Figure 10.**
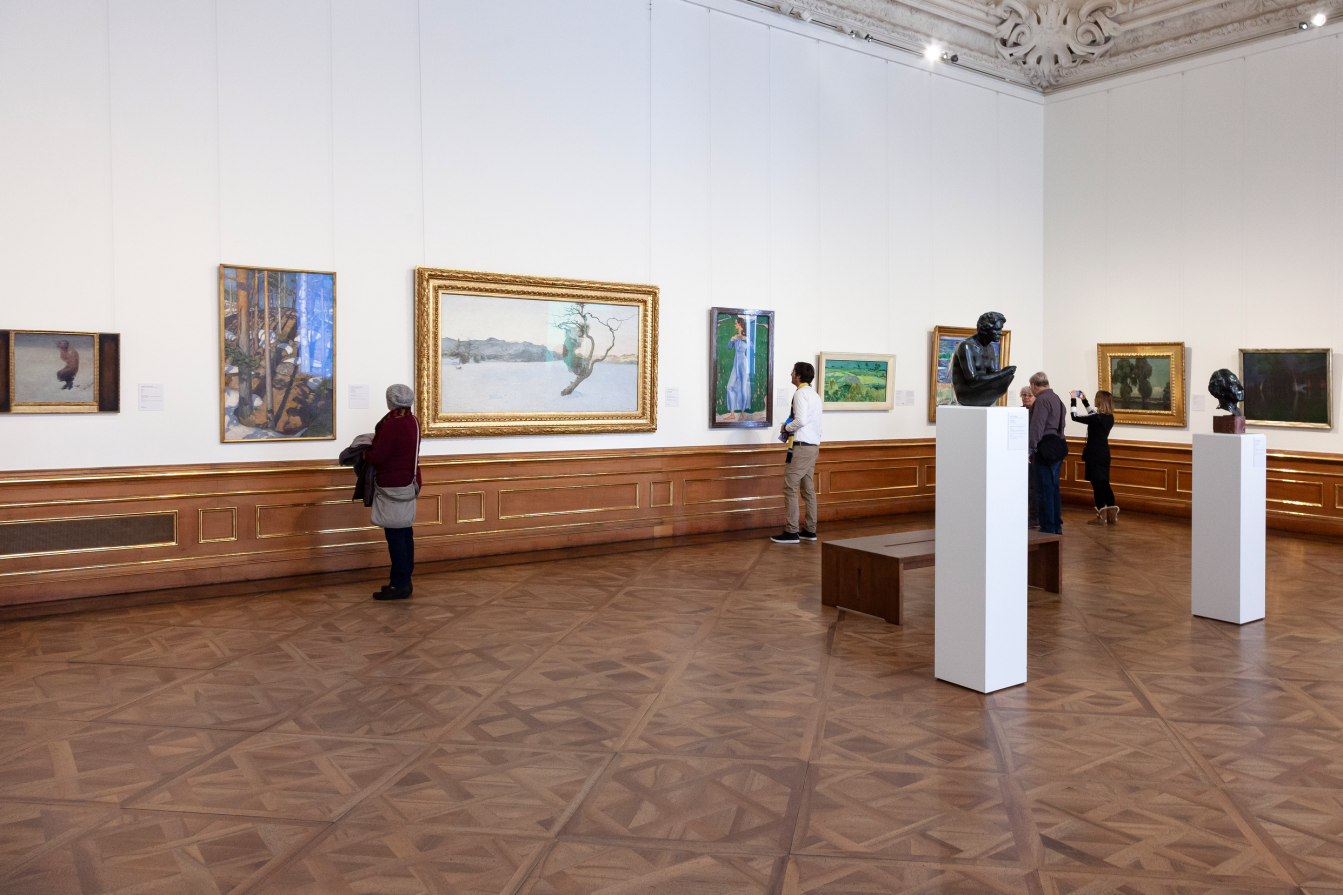
Installation shot with “Evil Mothers” (center) in BA. © Department of Art History, University of Vienna

While there is almost no difference of viewing time between BB and BA for “The Kiss,” two other artworks, “The Evil Mothers” (BB: *M* = 21.03 sec, *SD* = 18.66; BA: *M* = 30.58 sec, *SD* = 33.39) and “Judith” ” (BB: *M* = 24.44 sec, *SD* = 21.68; BA: *M* = 30.30 sec, *SD* = 31.02), experienced the greatest display effect in terms of increased mean viewing times (see Figures 7, 8, 9 and 10). Both of them are now located in the Secession room and aligned with more works on the wall. This new group presentation would not have led us to assume that more attention would be given to the two artworks. In BA, however, “Judith” was intriguingly more often talked about, with forty-three out of fifty participants referring to the artwork in their exhibition reflections (as opposed to only twenty-three in BB). Apart from “Judith” being often described as a famous Klimt painting, depicting an attractive woman with golden ornaments, the artwork’s interpretation deepened in BA with references to the biblical story of Judith and Holofernes or a general “gender trouble”, as indicated in the caption.

Additionally, the sight-line positioning of “Judith” next to the sculpture “The Nymph” and in visual relation to the “Portrait of Therese Bloch-Bauer” led visitors to notice and refer to stylistic similarities in the proud and/or seductive depictions of the female subjects. While many visitors were familiar with “Judith” beforehand (BB: 60.55%, BA: 74.67%), this was not the case with “The Evil Mothers” (BB: 10. 09%, BA: 3.33%). Here, the content analysis of the subjective mappings foregrounds the general attractiveness of the painting in both BB and BA. On the one hand, this means that the painting with its bright landscape, trees, and (hidden) women as well as babies stimulated “close looking.” On the other hand, the ambiguous title, “The Evil Mothers,” and the subtly suggested topics of motherhood and abortion stimulated further thoughts. In BA—with a mean viewing time increase of about ten seconds—this experience of a “double ambiguity” in the painting’s style and content led to far-reaching interpretations and strong emotional reactions.

### Time spent looking at art and reading text

Analyzing the time that visitors spent looking at the art and the accompanying labels (see Figure 10 as well as Tables A3 and A4 in the Appendix), we note a large shift from BB to BA: The mean total viewing time (including artwork and label) increased from 17.73 sec (*SD* = 21.96) to 26.14 sec (*SD* = 26.04) for the thirteen artworks that were presented in both settings. The introduction of new identification labels, and—in the case of four works—also captions with contextual information (as described in Materials), clearly proved beneficial towards mean total viewing times, with an increase of 47.43%.

This heightened attention pattern is, not surprisingly, even more evident for the four artworks that were given additional captions (see Table 5). Here, the mean total viewing time even increased (from BB to BA) from 29.25 sec (*SD* = 29.64) to 45.26 sec (*SD* = 45.63). This is an increase of 54.75% in total viewing time, with an even more impressive rise—a quintupling, in fact—in reading time of 538.48%. The introduction of these interpretive labels, we conclude, invited visitors to spend more time with those artworks, adding slightly to the viewing time and impressively to the reading time, as visitors accepted the new offer of (con)textual information.

**Table 5:** Viewing times in seconds for the 4 artworks and their labels in BB and BA (with additional captions)

	Belvedere Before			Belvedere After		
	Artwork	Label	Artwork & Label	Artwork	Label	Artwork & Label
	Mean (*SD*)	Mean (*SD*)	Mean (*SD*)	Mean (*SD*)	Mean (*SD*)	Mean (*SD*)
Kiss	56.94 (*38.57*)	1.70 (*3.33*)	58.64 (*38.71*)	57.92 (*48.54*)	14.09 (*21.61*)	72.01 (*57.84*)
Judith	24.44 (*21.68*)	1.69 (*2.04*)	26.13 (*21.90*)	30.30 (*31.02*)	16.51 (*17.51*)	46.80 (*39.97*)
Josef Lewinsky	13.26 (*11.45*)	3.05 (*2.47*)	16.31 (*11.97*)	18.15 (*24.05*)	14.74 (*18.27*)	32.89 (*38.58*)
Plain Auvers	13.15 (*12.69*)	2.77 (*3.67*)	15.91 (*14.21*)	15.84 (*18.20*)	13.50 (*16.33*)	29.35 (*29.64*)
Total	26.95 (29.61)	2.30 (3.07)	29.25 (29.64)	30.55 (36.36)	14.71 (18.43)	45.26 (*45.63*)

In addition, the room texts exhibited in each space were generally well received by visitors, with mean viewing times of 16.85 sec (*SD* = 22.87) for the first room text, 21.32 sec (*SD* = 25.05) for the second, and 24.69 sec (*SD* = 24.27) for the third. We note an incremental increase in thematic interest from the first room text, “Vienna around 1900: All the World’s a Stage,” to the second, “The Secession,” and again to the third, “Gustav Klimt.” Here, the text about the popular artist was read by more visitors and for a longer time, with a median viewing time of 24.32 sec (compared to 4.98 sec for room text 1 and 9.17 sec for room text 2). Moreover, the high standard deviation in reading time indicates that reading patterns varied widely among visitors. Analyzing these types of readers and deciphering the combined patterns of looking at artworks, reading text, and meaning-making, is a current subject of our further investigations.

The generally positive response to the additional text in BA is mirrored in the questionnaire, with visitors in BA ( *M* = 4.63, *SD* = 2.05) indicating that they would have liked more textual information to a lesser extent than did visitors in BB (*M* = 5.43, *SD* = 1.71; *F*(1,257) = 11.13, *p* = .002, generalized eta-squared = 0.04). In order to determine whether any of the visitors’ personal characteristics (gender, age, level of art interest, being a tourist or not, visiting alone or with others, visiting for the first time) could predict viewing and reading time in general, we ran a series of linear regressions over the entire sample of participants. Of these, only “interest in art” significantly predicted (longer) viewing time; however, the level of art interest only predicted viewing time for artworks (*R2* = .06, *F*(1,98) = 6.29, *p* = .01, *B* = 6.70) and for total viewing time (*R2* = .05, *F*(1,98) = 4.76, *p* = .03, *B* = 9.68), but not for time spent reading text ( *R2* = .00, *F*(1,98) = 0.00, *p* = .97, *B* = 0.05).

## Discussion

With the redisplay of the permanent collection of the Austrian Gallery Belvedere in spring 2018, our study seized the unique opportunity to analyze viewing behavior before and after a museum’s rearrangement. This endeavor was guided by the open research question: “How does the display influence the way people see and experience art in a museum?” Based on 1) the overall increase of viewing times for the artworks, 2) extended reading times, and 3) visitors’ deeper engagement with the artworks in their exhibition reflections after the museum’s rearrangement, we conclude that the display does, indeed, make a difference. It seems that the Austrian Gallery Belvedere delivers on its advertising claim that the Upper Belvedere could be experienced anew (83). This display effect is not mono-causal but related to a range of factors or powers that interact as competing influences in the authentic setting of the exhibition, where visual, material and social contexts intermingle. These four identified powers, i.e. the display, the artwork, the art form, and the person, guide the following discussion, also indicating the limits and prospects of our work.

### The power of the display

First of all, the display effect on viewing patterns depends on what visitors are given to see: In a rather classical art exhibition such as this, the objects presented were mainly artworks. The general distribution of attention—looking at art at 57.27% in BB and 48.10% in BA—suggests that looking at art is *the* main visual activity and the dominant visual task performed when spending time in an art exhibition. In addition, the distribution of attention in BB versus BA shows that introducing just one more ingredient—interpretive labels—changed the time spent looking at art and reading text from a rough 8:1 to 2:1 ratio. At first glance, this increased interest in the texts could be interpreted as a sort of “distraction” from the art. But as the total times show, this is not so: Visitors did not spend less time on the artworks but more time with them looking *and* reading. This is in line with the timing and tracking conclusion that visitors with longer dwell times—such as in BA spending one minute with 21.41 m^2^ vs. 26.26 m^2^ in BB, compared to an average Sweep Rate Index of 28 m^2^/min from a time database of more than 100 exhibits—tend to do more things rather than fewer things in depth (33, 34). This conclusion is also supported by the mean viewing times of the thirteen artworks that were shown in both conditions, where twelve out of thirteen received more attention.

The “power of information” as part of the “power of the display” greatly contributed to the success of the BA presentation. Here, we observe a significant shift of attention, the total viewing time including the artworks and the labels increasing from 17.73 sec ( *SD* = 21.96) to 26.14 sec (*SD* = 26.04) for the thirteen commonly presented artworks and from 29.25 sec (*SD* = 29.64) to 45.36 sec (*SD* = 45.63) for the four artworks that had additional interpretive labels. The captions with information on those works’ contexts, themes, and styles worked especially well as stimulating gestures that signify (as the artworks might say) both “look at me” and “read about me.” As visitors clearly followed this invitation to receive more contextual information, we conclude that interpretive text is a simple but very effective tool (from the curatorial and institutional side) of directing attention. The addition of text also proved to be beneficial towards visitors’ meaning-making, extending the depth and range of interpretations, as exemplified in the case of “Judith.” This value of interpretive text (rather than only identification labels) confirms that “part of the pleasure derived from looking at a painting stems from making a successful interpretation” (84). Long-standing evaluation work furthermore attests that labels that “contain concrete visually referenced information” foster combined reading and looking patterns, plus social activities such as pointing and talking (80). Consequently, we suggest that museums employ the label communication tool more consciously in order to enable multimodal and social encounters with artworks and other visitors. Another suggestion is to equip more artworks with additional captions, not only those that are already famous or from famous artists. This could also work towards visitors’ individual value-ratio choices in exhibitions in terms of the costs (time and effort of reading) they are willing to invest to obtain the benefit (such as contextual information and ideas for interpretation) in specific situations (35).

### The power of the artwork

The “power of the display” as influential presentation mode is, however, very much counteracted by the “power of the artwork.” No matter how the artworks are presented, a few specific artworks will always be in the focus of attention. It is noteworthy that visitors systematically spend very different amounts of time with different artworks, i.e. from the highest of almost one minute (BB: *M* = 56.94 sec, *SD* = 38.57; BA: *M* = 57.92 sec, *SD* = 48.54) to the lowest of a few seconds (BB: *M* = 4.91 sec, *SD* = 5.94; BA: *M* = 2.10 sec, *SD* = 4.73). In addition, as shown by the ranking of the thirteen artworks that were presented both in BB and BA, it is the same group of artworks that attracts either higher or lower attention time. This insight substantially expands our understanding of typical art viewing times, since these have previously been tested with fewer, famous or well frequented works only (30–32). Most often, these “attention magnets” are commonly referred to as “highlights” (as on the Belvedere museum’s website https://digital.belvedere.at/highlights) or, in tourist terminology, as “must sees and dos” (as on the travel blog shorturl.at/lMR27). This also means that the majority of visitors have seen them before, at least online. Following the spoken words of visitors, we suggest to name these “of course” artworks, as they are “of course” intensely looked at and strongly remembered.

In our set of artworks, “The Kiss” best demonstrates this “of course” status, joined by other works by “of course” artists such as Gustav Klimt, Vincent van Gogh, and Edvard Munch. This proves that fame and familiarity strongly influence our viewing behavior and enhance “the power of the artwork”. For the world-famous “The Kiss”, we detected the highest mean viewing time of almost one minute, comprising the highest mean number of 28 looks at the artwork per visitor, measured in both settings. This pattern of repeated returns and cumulative viewing time was already observed in a study with Gerhard Richter paintings (30), but without specification of the number of looks as the tracking was done manually. A comparison between the high viewing time for and the numerous looks at “The Kiss” with the subjective mapping statements, however, reveals that this time focus cannot be directly linked to liking the artwork or having had a fulfilling viewing experience. Partly, we were also able to detect ambivalent reactions to this work, being so well known in popular culture. On the negative side, the work’s celebrity combined with high expectations led some visitors to disappointment, disinterest or even distinction from the “masses” in the more or less crowded viewing situation on site. On the positive side, popularity led to excitement of some visitors, who could finally see the original or “the real thing” (bb-s010), following a path of (visual) anticipation. In the case of “The Kiss,” we can thus corroborate the statement that “time is not quality” (85, in their critique of Serrell’s timing and tracking indices) *per se*. Clearly, it depends on the specific exhibition situation and visitors’ individual perceptions whether investing viewing time and looks coincides with a positive art experience.

In contrast, the examples of “Judith” and “Evil Mothers”—works that received the highest viewing time increases after the museum’s rearrangement— show that spending more time can come with an increase in the quality of the art experience. Here, “the power of the artwork” was enhanced by “the power of the display” in the new curatorial presentation. Our analysis demonstrates that it is the combination of eye-appealing style and thought-provoking story that these ever-strong artworks revealed even more to visitors when they spent more time with them. In BA, the already famous “Judith” even became the “star” of the Secession room due to this heightened attention pattern. To the work’s advantage, it is now the only one by Klimt in the Secession room; it “seduces,” as it were, the viewer’s attention with the character’s own gaze and her story. This focus is supported by the information concerning the Judith and Holofernes story that is offered in the newly added interpretive label and the possible comparison with other female portraits in the Secession room. “The Evil Mothers” by Giovanni Segantini demonstrates that artworks that are not as famous or previously familiar to visitors also have the potential to attract attention. Despite its relative unfamiliarity, “The Evil Mothers” still ranked high in BB and even higher in BA, where it is the second-longest looked-at artwork after “The Kiss.” The difference we note between the two display conditions is how close the looking was and how far the interpretations went, as “The Evil Mothers” invited visitors to delve even more into visual details and to think about its ambiguous title and story in BA. This quality effect, however, could not be attributed to specific display changes referred to in visitors’ statements. Possibly, this effect was simply enabled by the availability of seating placed directly in front of the artwork, proving to be beneficial to viewing times (31).

### The power of the art form 

Looking at the lower rankings in viewing times, we additionally identified the “power of the art form” as an influential factor on viewing behavior. All three sculptures in BB and the seven in BA ranked at the lower end of the scale; the mean viewing time for sculptures amounted to less than half of the time for paintings in both settings: 6.00 sec (*SD =* 9.43) versus 16.67 sec (*SD =* 21.97) in BB and 5.55 sec (*SD =* 10.69) versus 17.27 sec (*SD =* 22.55) in BA. A possible explanation for this striking dominance of paintings over sculptures might be visitors’ expectations. As earlier research has shown, the influence on “entrance narratives” is extremely determinant in what people expect to see and, in consequence, are not only willing to see but will have seen when they leave the museum (44). In the case of the permanent collection of the Austrian Gallery Belvedere, expectations are very much bound to the art form of painting, especially when they “come for a Kiss” (according to the museum’s marketing campaign) or other of Klimt works. We assume that this pattern might be different when visitors expect to see sculpture primarily and not as part of an “extra” or a “side show.” Moreover, the rather lateral position of sculptures in BA, being positioned close to walls or the pathway, enhanced this peripheral perception. This was especially true for the “Crouching Woman” by Klinger as the only artwork among the thirteen presented in both settings that was looked at shorter after the museum’s rearrangement: While in BB one almost stumbled upon her “in the center [...] as a sculpture in a room of paintings” (bb-s030), in BA one can simply pass her by without the merest glance. This dominance of painting over sculpture contrasts, in part, with re-hanging experiments of the eMotion project, where sculptures evoked strong effects in the “force field” of the exhibition, even withdrawing attention from works on the wall. However, this effect depended on the size of the sculpture and its central position in the middle of the room (37).

### The power of the person

An important factor for the museum experience are the individual histories and psychological processes that visitors bring to the museum (35). This dimension can be regarded as “the power of the person.” In our study, however, we were able to prove that neither age, gender or the fact of being a tourist influenced viewing times. Regarding possible effects of being a tourist, however, our dataset might be misleading, since tourist visitors were overrepresented. The only variable that positively affected artworks and total viewing times was found in the level of art interest. In psychological theories of empirical aesthetics, this variable is regarded as central and often discussed in relation to art knowledge as the two main components defining art expertise (50, 51, 86). More importantly, art expertise as a determinant of aesthetic experiences is emphasized by all major psychological theories, claiming that one’s interest and knowledge about art changes one’s orientation towards art. This affects not only the cognitive, higher-order level of processing of art; expertise has also been shown to change the evaluation of art more generally, as people with higher expertise generally appreciate art more (87).

At the same time, empirical data clearly demonstrates that art interest and art knowledge are two distinct dimensions (77). For reasons of practicality, i.e. the length of the questionnaire, our study included the art interest scale but not the art knowledge scale of the VAIAK questionnaire. However, the art interest scale does not only refer to subjective interest items but also asks precisely about behavior that would indicate an interest in art (e.g., how often people visit museums). Thus, people who report that they often visit art museums (or engage in other related behavior) would be expected to also spend more time in viewing art in the museum, since one is obviously more likely to pay more attention to things one is more interested in (88). It is noteworthy, however, that in regard to reading the texts that were available in the rooms, art interest had no effect on the time spent on them.

## Conclusion

In conclusion, we note that the redisplay of the collection and the rearrangement of the museum had a measurable impact on how the artworks were viewed and experienced. Of course, unlike laboratory experiments with controlled manipulation of variables, it is not possible in such a field study to clearly ascribe changes to a specific factor. In the museum, indeed, the “power of the display” interacts with the “power of the artwork”, the “power of the art form,” and the “power of the person”. However, the innovative combination of MET with other analytical methods did provide significant insights into these power relations.

The limitations of our study are consequently linked to the specificity of a field study where validity takes precedence over reliability. Far from entering into a lab versus museum debate, we suggest consciously opting for one or the other direction, ideally combining findings from both controlled and unconstrained studies as well as combining (mobile) eye tracking with other methods. With respect to MET data analysis, we hope to soon overcome the limitation of having to rely on manual data annotation. A data analysis program based on object recognition and machine learning would save analysts from spending countless hours in data preparation and enable more complex single-object data analysis beyond the measuring of viewing time (e.g., viewing behavior with regard to form and content of specific artworks) as well as inter-object-related data analysis (e.g., gaze trajectories between artworks and people in crowded situations).

Powerful and accessible MET data analysis programs would also facilitate further analysis of the data collected in the “Belvedere Before and After” study moving beyond the scope of this paper to present first general results. Future work, departing from phenomena encountered in this exploratory study, will specifically investigate seeing in relation to other practices. This includes seeing and reading (to explore gaze patterns between image and text as well as corresponding meaning-making processes); seeing and using a smartphone (to empirically frame the impact of this digital tool on contemporary museum practice); as well as seeing and moving in an exhibition space (to investigate the numerous viewpoints in relation to the art forms of painting and sculpture). In these ways, then, we aim to further understand the museum gaze from multiple perspectives.

## Ethics and Conflict of Interest

The authors declare that the contents of the article are in agreement with the ethics described in http://biblio.unibe.ch/portale/elibrary/BOP/jemr/ethics.html and that there is no conflict of interest regarding the publication of this paper. The study was approved by the ethics committee of the University of Vienna, participants signed a detailed consent form, and no visitors under 18 took part.

## Acknowledgements

We would like to cordially thank the whole team of the Austrian Gallery Belvedere with its Scientific Director, Stella Rollig, for the collaboration and especially Margarete Stechl for the daily support. Our heartfelt thanks also go to Anna Cornelia Barbulesco, Jane Boddy, Max Douda, Anna Fekete, Judith Herunter, Sarah Hübler, Rebeka Jovanoska, Jisoo Kim, Katrin Kopp, Marthe Kretzschmar, Rosita Messmer, Kristina Miklosova, Adrian Praschl-Bichler, Rebekah Rodriguez, Stephanie Sailer, Rosa Sancarlo, Berna Selin Sayin, Hamida Sivac, Mariette Soulat, Julia Starke, Clara Swaboda, Magdalena Syen, Daniel Teibrich, Veronika Vishnevskaia, and Sophie Wratzfeld for their valuable help in data collection and data preparation. This research was supported in part by the Austrian Science Fund (FWF, grant P25821 and P27355) and the Vienna Science and Technology Fund (WWTF, grant CS15-036). Open access funding was provided by the University of Vienna.

## Figures and Tables

**Figure 1:**
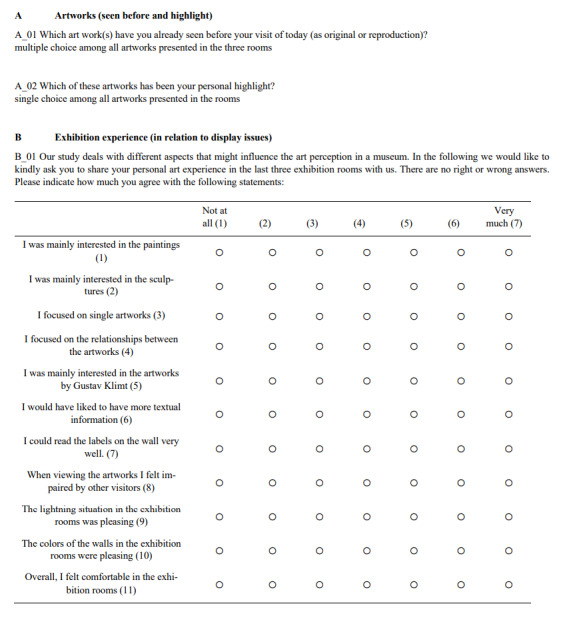
Question A/Question B

**Figure 2:**
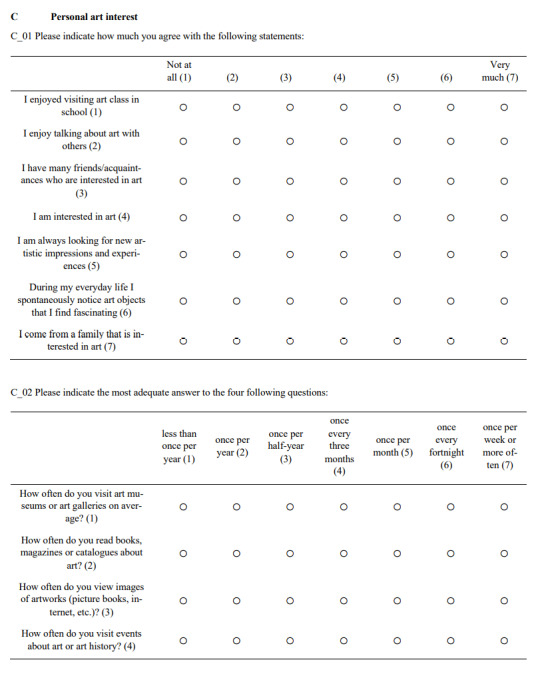
Question C

**Figure 3:**
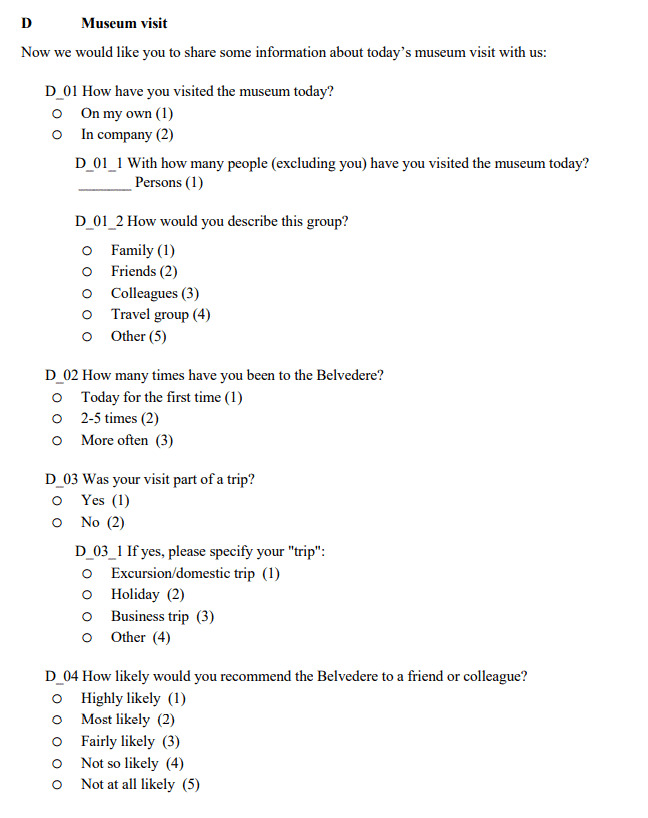
Question D

**Figure 4:**
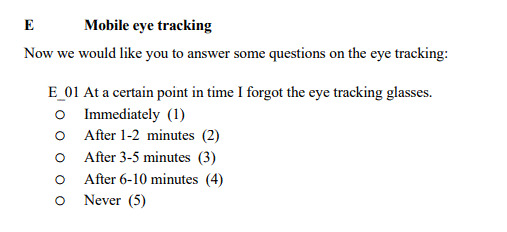
Question E_01

**Figure 5:**
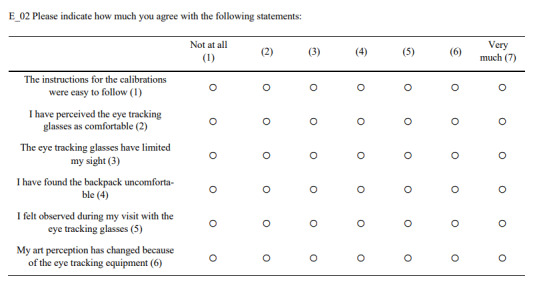
Question E_02

**Figure 6:**
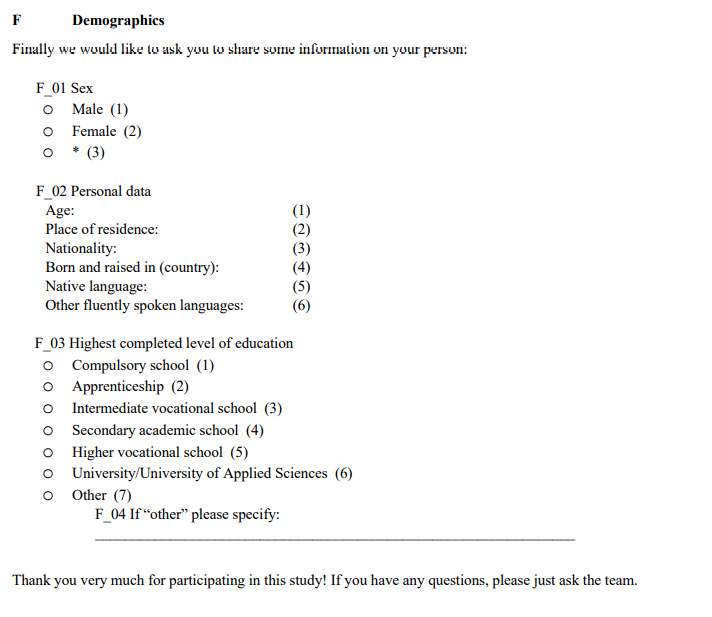
Question F

## Appendix

### Tables

**Table A1:** List of artworks in BB (in hanging order)

Belvedere Before			
Artist	Title	Abbreviated title	Year
Bernatzik, Wilhelm	Pond	Pond	c. 1900
Khnopff, Fernand	Calm Water (The Pond of Menil)	Calm Water	1896
Moll, Carl	Twilight	Twilight	1900
Gallén-Kallela, Akseli	Early Spring	Early Spring	c. 1900
Segantini, Giovanni	The Evil Mothers	Evil Mothers	1894
Orlik, Emil	Dachstein	Dachstein	1904
Moser, Koloman	Self-Portrait	Self-Portrait	1890
Van Gogh, Vincent	The Plain of Auvers	Plain Auvers	1890
Munch, Edvard	The Painter Paul Herrmann and the Physician Paul Contard	Painter Physician	1897
Rodin, Auguste	Eve	Eve	1881
Schindler, Emil Jakob	Pax	Pax	1891
Klinger, Max	The Judgement of Paris	Judgement	1885-1887
Böcklin, Arnold	Sea Idyll	Sea Idyll	1887
Von Hofman, Ludwig	Largo (Sunset)	Largo	c. 1898
Khnopff, Fernand	Half-figure of a Nymph (“Vivien”)	Nymph	1896
Klinger, Max	Crouching Woman	Crouching Woman	1900-1901
Klimt, Gustav	Amalie Zuckerkandl	Amalie Zuckerkandl	1917/1918
Klimt, Gustav	The Bride	Bride	1917/1918
Klimt, Gustav	Fritza Riedler	Fritza Riedler	1906
Klimt, Gustav	Judith	Judith	1901
Klimt, Gustav	Josef Lewinsky as Carlos in Clavigo	Josef Lewinsky	1895
Klimt, Gustav	Lady in White	Lady White	1917/1918
Klimt, Gustav	Girlfriends (Water Serpents I)	Girlfriends	1904
Klimt, Gustav	Lady at the Fireplace	Lady Fireplace	1897/1898
Klimt, Gustav	Draft for the Allegory of Music (The organ player)	Allegory Music	1885
Klimt, Gustav	The Kiss (Lovers)	Kiss	1908/1909

**Table A2:** List of artworks in BA (in hanging order)

Belvedere After			
Artist	Title	Abbreviated title	Year
Makart, Hans	Dante and Vergil in Hell	Dante Vergil	1863/1865
Makart, Hans	Antique Sacrifice	Antique Sacrifice	c. 1880
Feuerbach, Anselm	Orpheus and Eurydice	Orpheus Eurydice	1868-1869
Böcklin, Arnold	Sea Idyll	Sea Idyll	1887
Klimt, Gustav	Josef Lewinsky as Carlos in Clavigo	Josef Lewinsky	1895
Klimt, Gustav	Marie Kerner von Marilaun as a Bride	Marie Kerner	1891-1892
Klimt, Gustav	Portrait of a Lady in Black	Lady Black	c. 1894
Klinger, Max	Crouching Woman	Crouching Woman	1900-1901
Friedl, Theodor	Cupid and Psyche	Cupid Psyche	c. 1890
Bernatzik, Wilhelm	Pond	Pond	c. 1900
Moll, Carl	Twilight	Twilight	1900
Hölzel, Adolf	White Poplars	White Poplars	1900
Munch, Edvard	Men on the Seashore	Seashore	1908
Van Gogh, Vincent	The Plain of Auvers	Plain Auvers	1890
Hodler, Ferdinand	Emotion	Emotion	1900
Segantini, Giovanni	The Evil Mothers	Evil Mothers	1894
Gallén-Kallela, Akseli	Early Spring	Early Spring	c. 1900
Von Stuck, Franz	Lost	Lost	1891
Minne, Georg	Woman Bathing	Woman Bathing	Undated
Klimt, Gustav	Judith	Judith	1901
Khnopff, Fernand	Half-figure of a Nymph (“Vivien”)	Nymph	1896
Jaschke, Franz	Donaulände in Summer	Donaulände Summer	1903
Kurzweil, Max	Portrait of Therese Bloch-Bauer	Therese Bloch-Bauer	um 1907
Rodin, Auguste	Gustav Mahler	Gustav Mahler	1909
Klinger, Max	Beethoven	Beethoven	1907
Klimt, Gustav	Schloss Kammer on Lake Attersee III	Schloss Kammer	1909/1910
Klimt, Gustav	Sonja Knips	Sonja Knips	1897/1898
Klimt, Gustav	Fritza Riedler	Fritza Riedler	1906
Gerstl, Richard	The Sisters Karoline and Pauline Fey	Sisters Fey	1905
Kokoschka, Oskar	The Visitation	Visitation	1912
Klimt, Gustav	Forester’s House in Weissenbach on the Attersee I	Forester’s House	1914
Klimt, Gustav	The Kiss (Lovers)	Kiss	1908/1909
Klimt, Gustav	Flowering Poppies	Flowering Poppies	1907
Rodin, Auguste	Eve	Eve	1881
Klimt, Gustav	Cottage Garden with Sunflowers	Cottage Garden	1907

**Table A3:** Viewing times in seconds for 13 artworks and labels in BB

N°	Belvedere Before	Artwork	Label	Artwork & Label
	Abbreviated title	Mean (*SD*)	Mean (*SD*)	Mean (*SD*)
1	Kiss	56.94 (*38.57*)	1.70 (*3.33*)	58.64 (*38.71*)
2	Judith	24.44 (*21.68*)	1.69 (*2.04*)	26.13 (*21.90*)
3	Fritza Riedler	23.86 (*16.16*)	2.20 (*3.13*)	26.06 (*16.66*)
4	Evil Mothers	21.03 (*18.66*)	2.41 (*3.01*)	23.44 (*19.94*)
5	Sea Idyll	15.66 (*12.81*)	1.93 (*2.44*)	17.59 *(13.71)*
6	Josef Lewinsky	13.26 (*11.45*)	3.05 (*2.82*)	16.31 (*11.97*)
7	Plain Auvers	13.15 (*12.69*)	2.77 (*3.67*)	15.91 (*14.21*)
8	Early Spring	8.70 (*8.78*)	2.47 (*3.26*)	11.17 (*10.62*)
9	Pond	7.34 (*5.81*)	2.00 (*2.94*)	9.35 (*6.72*)
10	Nymph	6.65 (*7.87*)	0.70 (*2.01*)	7.35 (*8.76*)
11	Crouching Woman	6.27 (*12.69*)	0.14 (*0.60*)	6.41 (*12.65*)
12	Twilight	5.38 (*6.05*)	1.67 (*2.20*)	7.05 (*7.19*)
13	Eve	5.09 (*6.78*)	0.00 (*0.00*)	5.09 (*6.78)*
	Total	15.98 (*21.07*)	1.89 *(2.76*)	17.73 *(21.65*)

**Table A4:** Viewing times in seconds for 13 artworks and labels in BA

N°	Belvedere After	Artwork	Label	Artwork & Label
	Abbreviated title	Mean (*SD*)	Mean (*SD*)	Mean (*SD*)
1	Kiss	57.92 (*48.54*)	14.09 (*21.61*)	72.01 (*57.84*)
2	Evil Mothers	30.58 (*33.39*)	4.75 (*5.01*)	35.34 (*35.78*)
3	Judith	30.30 (*31.02*)	16.51 (*17.51*)	46.80 (*39.97*)
4	Fritza Riedler	27.60 (*27.80*)	3.21 (*3.65*)	30.81 (*28.95*)
5	Sea Idyll	21.31 (*16.60*)	3.83 (*5.71*)	25.13 (*18.76*)
6	Josef Lewinsky	18.15 (*24.05*)	14.74 (*18.27*)	32.89 (*38.58*)
7	Plain Auvers	15.84 (*18.02*)	13.50 (*16.33*)	29.35 (*29.64*)
8	Nymph	11.53 (*18.16*)	3.35 (*4.17*)	14.87 (*21.67*)
9	Pond	11.01 (*21.71*)	3.18 (*3.85*)	14.19 (*23.97*)
10	Early Spring	9.47 (*9.40*)	2.67 (*2.61*)	12.14 (*10.78*)
11	Twilight	8.86 (*10.89*)	2.64 (*3.23*)	11.50 (*12.84*)
12	Eve	7.40 (*13.73*)	1.48 (*2.02*)	8.88 (*14.55*)
13	Crouching Woman	4.19 (*6.85*)	1.77 (*3.05*)	5.96 (*9.22*)
	Total	19.55 (*27.77*)	6.59 (*12.01*)	26.14 (*34.28*)

Questionnaire
